# Ultra Low Power Signal Oriented Approach for Wireless Health Monitoring

**DOI:** 10.3390/s120607917

**Published:** 2012-06-08

**Authors:** Stevan Marinkovic, Emanuel Popovici

**Affiliations:** Department of Electrical and Electronic Engineering, University College Cork, Cork 11111, Ireland

**Keywords:** wireless body area network, wake up receiver, mHealth

## Abstract

In recent years there is growing pressure on the medical sector to reduce costs while maintaining or even improving the quality of care. A potential solution to this problem is real time and/or remote patient monitoring by using mobile devices. To achieve this, medical sensors with wireless communication, computational and energy harvesting capabilities are networked on, or in, the human body forming what is commonly called a Wireless Body Area Network (WBAN). We present the implementation of a novel Wake Up Receiver (WUR) in the context of standardised wireless protocols, in a signal-oriented WBAN environment and present a novel protocol intended for wireless health monitoring (WhMAC). WhMAC is a TDMA-based protocol with very low power consumption. It utilises WBAN-specific features and a novel ultra low power wake up receiver technology, to achieve flexible and at the same time very low power wireless data transfer of physiological signals. As the main application is in the medical domain, or personal health monitoring, the protocol caters for different types of medical sensors. We define four sensor modes, in which the sensors can transmit data, depending on the sensor type and emergency level. A full power dissipation model is provided for the protocol, with individual hardware and application parameters. Finally, an example application shows the reduction in the power consumption for different data monitoring scenarios.

## 1. Introduction

Mobile (m)Health systems [1] are a fast growing research field that encompasses the use of mobile telecommunication and multimedia technologies within increasingly mobile and wireless health care systems. This field has emerged as a sub-field of eHealth and it is pushing the limits of how to acquire, transport, store, process, and secure both raw and processed medical data to deliver meaningful results. In particular, it offers the possibility of remotely monitoring patients and allowing them to participate in health care systems, a feature which may not have been possible in the past. Two of the main goals of this types of system are to improve the quality of care and reduce cost. The proposed framework addresses issues in both developed and developing nations where mobile communications technologies are becoming widely accessible.

A Wireless Body Area Network (WBAN) is a constituent part of mHealth systems and consists of multiple nodes located at different locations on the human body. A WBAN is a wireless network consisting of independent sensor nodes that are attached to the body surface, implanted in the body or around the body, to provide part of the infrastructure in pervasive ubiquitous systems. These devices can be combined with common devices such as personal digital assistants (PDAs), smart phones, and headsets to further provide a platform for applications such as unobtrusive healthcare, sports performance monitoring, ambient intelligence, entertainment, vital signs monitoring, as well as many other potential uses that utilise the physiological signals.

The location of a sensor mainly depends on the function of the sensor attached to the node. As a consequence, depending on the physiological parameters being monitored, one can have different sensors and different network topologies. Each individual sensor, or group of nearby sensors, is connected to the main mHealth bridge node wirelessly. The bridge node is often a more powerful device equipped with a larger battery than the sensor nodes. The wireless connectivity is important for multiple reasons, such as to reduce cost of care, to ease the sensor application and improve patient comfort, and, finally, to enable remote (e.g., at home) patient monitoring. Some examples of sensors include electroencephalogram (EEG), electrocardiogram (ECG), oxygen saturation (SpO2), accelerometer, global positioning system (GPS), temperature, microphones, mechanomyography (MMG), electromyography (EMG), blood pressure, *etc.*

**Figure 1.**
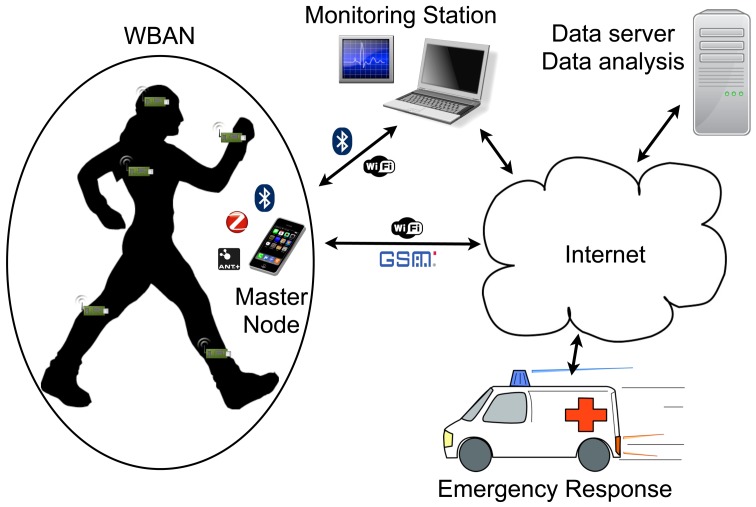
Example of WBAN integration.

Figure 1 shows the typical architecture of a WBAN sensor network and its possible integration with larger scale networks. The wearable wireless enabled sensors attached to the body take in the physiological readings, and together form the WBAN. These sensors can be implantable or on body, and can be of various types, such as temperature, accelerometer, ECG, EEG, *etc.*

The WBAN has one network coordinator or Master Node. This can be a dedicated device or any PDA or a smartphone. It acts as a gateway between the WBAN and the larger scale network. The master node relays the BAN information to a monitoring station that can provide further services through a Local Area Network or the Internet. If the master node is WiFi or GSM enabled, it can directly transfer data trough the Internet. This interface is already well developed and standardised; however, redundancy is required at this level for critical applications, as this is a single point of failure in the network.

The main research effort in the WBAN concept was to reduce the power consumption of a sensor node, while still keeping or enhancing its functionality. Low power consumption is paramount for these devices, since due to their application, the sensors have to be small to be wearable and unobtrusive. The miniaturisation leads to a requirement for reduced battery size, and thus the overall power budget gets reduced. For some medical applications such as implantable sensors/actuator devices, long lifetime requirements (10–15 years) for the sensor node may lead to energy harvesters to be employed, which puts further constraints on the available energy budget.

This work focuses on the reduction of communications power consumption in the WBAN sensor node by using novel communication methods that take advantage of the wireless wake-up functionality enabled by the our Wake Up Receiver (WUR) [2]. Implementation of the WUR has benefits in networks that do not have high data rates and traffic but need to have on-demand functionality with fast response times. As recent low power DSP processors have enabled low power signal analysis and pre-processing, WBAN sensors have increasing processing capabilities and can be split in two groups depending on whether they transmit only raw measured data or do DSP preprocessing. Some signals (ECG for example) contain more than one type of information about the body (ECG values, heart rate, respiratory rate, *etc*….) and the DSP enabled sensors can extract this information. This leads to lower communication data rates “over the air” where nodes transmit extracted physiological values instead of raw physiological signals. On the other hand, this increases the need for a network that is responsive and can handle the events to transmit these physiological values when they are available, computed, or demanded from an external monitor. We will present a communication method that takes this into account and, using the WUR, formalises the communication within a WBAN for overall power efficiency and prolonged sensor battery lifetime and independence.

## 2. Related Work in WBAN MAC Protocols

Most of the devices used in WBAN applications store the recorded data or transmit it to a monitoring station using IEEE 802.15.1 (Bluetooth) or IEEE 802.15.4 (ZigBee) protocols. These wireless standards are well documented and tested, but are not an ideal choice for the wireless BAN because they target more flexible networks and are used for longer transmission ranges. This makes them less energy efficient than protocols that are specifically targeting a BAN [3]. A comparison and optimisation of two popular WBAN technologies, Bluetooth and ZigBee, is given in the comparative study [4], in terms of design, cost, performance and energy efficiency.

As mentioned in [5] the long term health monitoring of patients requires low power techniques. Medium Access Control (MAC) protocols play a significant role in determining the energy consumption in wireless communication. Traditional MAC protocols mainly focus on improving bandwidth utilisation, throughput, and latency. However, they lack energy conserving mechanisms, which is one of the most important constraints of a WBAN. The main sources of energy waste are collision, idle listening, overhearing, and control packet overhead. MAC protocols maximise the network lifetime by controlling the aforementioned sources of energy waste. Some contention based protocols such as WiseMAC [6] and BMAC [7] use low power listening and preamble sampling techniques to reduce idle listening. Other protocols such as SMAC [8], TMAC [9] and PMAC [10] reduce idle listening by applying a synchronised schedule between the nodes. Contention-based solutions are not suitable for WBAN because most of the traffic is correlated.

Recent works have proposed TDMA-based MAC protocols for wireless sensor networks [11–14] and protocols especially for WBAN [15–19]. Some are directed by the type of transceiver used [15,16].

Newer WBAN protocols incorporate the concept of Wake Up Receiver (WUR) in their design. A Traffic-adaptive MAC protocol (TaMAC) for WBAN that improves energy efficiency by exploiting the traffic information (traffic-patterns) of the nodes is presented in [5]. Other solutions that incorporate WUR for energy efficiency is presented in [20,21]. These solutions are based on a fictional (concept) WUR, but prove that if presented with a WUR device, power consumption of any protocol can be significantly reduced.

We expand the work on wireless wake-up functionality protocols by including a developed and tested WUR, and present a formalisation in the context of Wireless Health monitoring.

## 3. Power Efficient Wake-Up Receiver for WBAN

There are three types of communication in a WBAN: on-demand, emergency, and normal traffic. On-demand traffic is initiated by the coordinator to acquire instantaneous real-time information, mostly for the purpose of diagnostic recommendations. This is further divided into continuous (data stream) and discontinuous communication. Emergency traffic is initiated by the nodes when a critical parameter exceeds a predefined threshold and should be accommodated as soon as possible. This kind of traffic is not generated on regular intervals and is totally unpredictable. Normal traffic is the data traffic in a normal condition with no time critical or on-demand events. This includes unobtrusive and routine signal monitoring.

Several techniques have been proposed to minimise the transceiver's duty cycle (the percentage of time during for which it is in an “active” state). However, while reducing the duty cycle helps to save power, it severely limits network flexibility. The transceiver's power consumption while listening idly on a channel can approach or even exceed power consumption during data transmission. Therefore, if there is a need for asynchronous events to be initiated by the network coordinator, considerable energy must be spent by individual sensors during idle listening. To avoid wasting energy, a WBAN requires an ultra-low-power wireless receiver which takes over the task of listening for asynchronous signals, allowing the primary transceiver to be shut down.

A typical deployment of a dedicated WUR in a wireless sensor would be as an additional circuit to an existing wireless transceiver. It should communicate with the sensor's microcontroller via some standard interface, and ideally re-use the transceiver's antenna and the communication frequency dedicated to the WBAN. A typical application will be in a WBAN network with ranges less than 2 m. There will be a dedicated device acting as a Master Node that will act as a network coordinator. This device would send wake up signals, and receive data packets from sensors.

The wake up receiver intended for a WBAN is expected to operate in the dense network environment. At any given moment some nodes will be communicating within a WBAN, while some may stay in a sleep mode, monitoring the channel for communication requests. Also, in the vicinity of a network there will be numerous high power transceivers and noise. The WUR must be robust to this ambient traffic and should avoid waking up the sensor on signals intended for the neighbouring nodes, as well as on the ambient noise.

From a functional perspective, a WUR is not as restricted as the standard receiver regarding bit error rate and data rate. The metric of interest is the probability of detected wake up signals, because re-sending them increases power consumption for the transmitter as well as the latency. A second metric is the reduction of false alarms. A false alarm is also costly from a power perspective because the sensor is activated needlessly.

**Figure 2.**
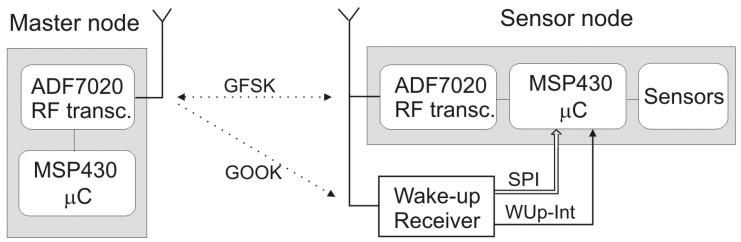
Wake Up Receiver Application.

A low power semi-passive wake up receiver was developed and tested [2]. The receiver application can be seen in Figure 2. It is based on an envelope detector, used for the OOK demodulation. A charge pump (two-stage voltage doubler-multiplier) is used as an OOK signal envelope detector, as first presented in [22]. This demodulated signal is fed to a data slicer, which raises the demodulated digital signal to voltage levels that can be used by later digital circuitry. The comparator threshold is adaptive, rather than constant, and is determined by the wake-up signal strength. This digital signal is then classified using the passive circuitry in order to detect the “intended” wake up signal and to reject the random toggling due to noise or nearby wireless communications. The classification is done by detecting the wake-up preamble if it is generated at the expected OOK data rate range. The next part of the circuit is a Pulse-Width Modulation (PWM) decoder and SPI adapter. This part of the circuit first decodes the PWM encoded signal and generates SPI compatible signals for data transfer to the processor.

The architecture is power efficient since most components constituting it are passive. The only active components are the data slicer (comparator) and the SPI adapter. Due to the simplicity of this circuit, the power consumption is kept very low. The basic functionality is achieved by using an envelope detector as a simple and low power wireless receiver, and building simple active circuitry around it to raise the voltage levels, reject false wake up signals, and make the output SPI compatible.

Measurements show that the static current consumption of our WUR, while listening on a wireless channel, is 180 nA at 1.5 V. Therefore, the static power consumption (when listening on a channel) is 270 nW. This, with the dynamic power of 1.75 nJ/bit and sensitivity of −51 dBm for 5.5 kbit/s data rate, satisfies the energy consumption/range requirements for WBAN. The data rate is sufficient for a 10–15 byte wake up packet length containing information for addressing the sensor node to be woken as well as some additional information and a CRC field.

## 4. Signals in the WBAN

Many new methods are being developed for low power DSP processing of physiological body signals. These allow some pre-processing to be done directly on a sensor node, so that only useful data needs to be transmitted instead of the whole raw signal. Some examples for EEG data preprocessing on a sensor node are presented in [23,24]. EEG data can be preprocessed in order to extract events such as when a seizure happened, the type of seizure, and how long it lasted. Other work that shows how an ECG signal can be preprocessed to output the respiration rate is presented in [25]. The ECG is used to extract “common” values such as heart rate, and heart condition. Other studies suggest that similar values can be extracted from an SPO2 sensor in addition to the main measurement of oxygen saturation extraction [26]. Therefore, data can be preprocessed to get the actual physiological quantities at the sensor, where the sensors can also be “smart” and notify the coordinator if some values go above or below defined thresholds, or some event occurs. Also, the sensor can measure multiple quantities with a certain accuracy, but, more importantly, one quantity (pulse for example) can be measured using different sensors.

Therefore, sensors and medical devices with an added microprocessor and wireless connectivity can be split into three groups:

1. Sensors that stream data
  They have high sample rates and are used for data transfer on demand (ECG, EEG, EMG, SPO2, *etc.*).
2. Sensors that are used for constant patient monitoring
  They usually have very low sample rates, and in general would be sleeping for extended periods of time, but regularly waking up, measuring some value and sending it. Examples of those would be temperature, glucose level, *etc.*
3. DSP enabled sensors
  Sensors that can transfer data on demand, but can also perform local DSP and send values, such as the sensors in category 2. For example, ECG or SPO2 sensors can stream data, but can also give the status for heart rate or respiration rate.

The sensors from groups 2 and 3 can generate an event if a value passes a predefined threshold.

Protocols intended for medical applications would deal mostly with notifications and actual information on the extracted parameters as well as the random “events”, instead of transfers of large quantities of data to the central processing system. But, they would also have to stream data occasionally on demand. Therefore, we will analyse the proposed WUR in medical settings as a method of keeping these networks responsive to the random events and signals as well as to cater for higher volume data transfer.

## 5. Signal Oriented—Event Driven Approach

We will consider a WBAN as a one-hop star network, built around a Master Node (MN). We use this network topology rather than multi-hop, since idle listening is reduced, and thus the power consumption is lower. The MN uses WUR enabled protocols for intra-BAN communication and some other wireless communication methods for the data transport to the monitoring stations. The MN usually has less power constraints and is equipped with a larger battery. The nodes can be dedicated gateways for the transmission over longer distances, or they can be a PDA/smartphone device which can provide data analysis/DSP, and some basic monitoring as well as acting as a data relay.

If the protocol is to be used in medical settings for patient monitoring, or in home based monitoring systems, it should be signal-oriented instead of sensor-oriented. In such cases, the time slots are assigned to physiological signal information, and then to the sensors that can provide them. This is to accommodate possible smart sensors that can extract multiple information from the signals, to provide a complete m-Health system, as summarised in [1].

Each wireless sensor in a WBAN should have a set of services it can provide. During the formation of a WBAN network for a patient, the list of these services for each sensor should be sent to the network coordinator. The network coordinator then assigns the wireless resources to each sensor, specifies then information that sensor needs to provide and the rate at which this information is to be updated. This would vary depending on scenario, and the type of patient monitoring needed.

Looking from the TDMA perspective, every time slot has a physiological quantity assigned to it, and the preferred sensor from which this quantity is obtained. For example, if pulse is needed, the MN assigns a time slot for all communications regarding this parameter. Then it sends a wake up signal and a command to the address that is used by the SPO2 sensor, to measure the pulse. If the sensor does not respond, the MN addresses an alternate sensor (such as a DSP enabled ECG), asking for the same value, before eventually concluding that the required signal is unavailable. Also, if streaming of the data is necessary, the MN addresses the sensor, and assigns a number of timeslots, depending on data rate and required latency (generally, it would give the number of time slots required for streaming, and some reserve timeslots).

From the sensors perspective, there can be four modes of operation for a sensor:

1. *Disconnected*—The sensor state after reset. It is in a deep sleep mode, waiting to be assigned to a WBAN.
2. *Stand-by*—The sensor is in a deep sleep mode, but is paired with a MN, waiting for a wake-up signal to change its state, to get assigned to a channel.
3. *Monitoring*—The sensor is in a stand-by mode, but it wakes up once or periodically, with a period assigned by the MN (the measurement period), to send a measured or computed value. Depending on the signal, it might be necessary to switch on the sensor earlier.
4. *Stream*—This might be available for some sensors. The sensor is on, collecting data, packaging the raw samples and sending data to the MN.

While the sensor is in a stream mode, data is exchanged frequently, and the sensor is responsive to sudden changes or requests from the MN (since it is online). For the other modes, the sensor will be off most of the time, with infrequent data transmission, waking up only to transmit measured data. In order to be responsive, it must monitor a channel, and keep the communication with some predefined time interval between the beacons. The wake up radio has great potential in these modes of operation.

The Master Node can change the state of the sensor, using a wake up packet and a command packet, or alternatively using an ACK packet, if the sensor is active during the time slot. The standby mode is useful when the data only needs to be gathered infrequently by medical staff.

As far as MN is concerned, there are three states of operation. Depending on the medical practices there can be various sub-modes. But all of them can be split in three basic modes:

1. *Stand-by*—The network is organised, and the sensors are paired with a MN, but there is no communication activity. The network is in a ready state to quickly start obtaining data if needed.
2. *Normal monitoring*—All the sensors are in stand-by mode and paired. The MN is not sending beacons, in order to save energy. At regular intervals, the MN searches for a free channel, starts sending beacons and wake up packets, collects the data from the sensors, and frees up the channel.
3. *Emergency—On demand monitoring*—The MN is sending beacons in a channel or with a predetermined frequency hopping scheme. This would usually mean that there is an emergency, or somebody has requested monitoring or a channel stream.

Changing the network operation from stand by to monitoring or vice versa would usually be initiated by medical staff or some predetermined scenario-schedule. Triggering emergency monitoring can be done by medical staff, if the MN detects health deterioration from normal, or possibly by the “smart” sensors or sensor groups that can immediately detect dangerous values in the signals they are monitoring.

### 5.1. Protocols in Signal Oriented-Event Driven Environment

Popular protocols can be used in these scenarios, in conjunction with a WUR, in order to provide an efficient WBAN network for medical applications. The WUR can be used for a quick transition between the sensor states (stand-by, monitoring, *etc.*) in the network, without the need for the main transceiver to be on. The signal approach can be realised in higher OSI layers of the protocol, and this can be protocol independent. The protocols do not need any modifications in order to be efficient with a WUR. The WUR signals have different modulation and they can operate in a different frequency band (although this is not recommended due to antenna and matching issues). Ideally, there can be a frequency band reserved for wake up packets, since the rate of occurrence of these packets is relatively low and WUR sensitivity is not high.

The signal approach is realised in the application layer. The application layer of the sensor contains the services that the sensor can provide. This should be protocol independent. During network forming, which is done by a standardised method for each communication protocol, this list of services is obtained from all of the sensors within a network, and depending on the required scenario, the sensors are given the task of measuring certain values. In order to avoid a situation where the sensor would be constantly in the disconnected mode, searching for a network, which is power consuming in most of the described protocols, we consider a broadcast wake up packet, which is used for the sensors in off mode and not assigned to any network to wake up in a search for Master Node using the pairing process defined by the protocol. If the sensor is not paired with a MN after some time out period, it would return to the off mode, leaving only the WUR to be active.

**Figure 3.**
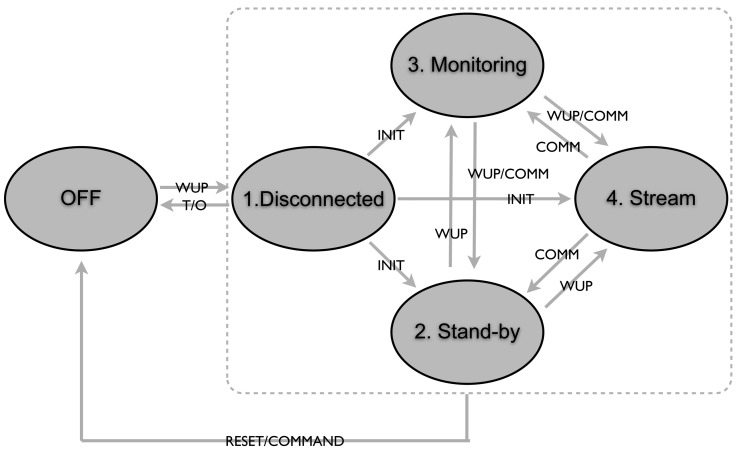
Chart of the sensor states for an event driven environment with possible transitions between them.

After this initial process, the sensors go into the required state and they can change states as illustrated in Figure 3. The transition between states are:

- INIT—Transition from the unattached state to being connected. It is done according to the protocol.
- WUP—Transition initiated by the Wake Up Radio, followed by a command for transition.
- COMM—Transition initiated by the Master Node using the regular receiver while acknowledging the packet or in a beacon.
- RESET—Transition initiated by the Master Node using the WUR or regular communication, or initiated by an external reset.
- T/O—Transition to Off after a certain time out period.

The wake up packet can be a network address assigned during network forming, or a hardware MAC identifier as defined in a protocol, for better robustness and better reliability in the case of the multiple BANs operating in the vicinity. In the case of Bluetooth, a MAC-48 identifier can be used, or in case of ZigBee, EUI-64. The wake up packets can optionally contain some other defined commands and identifiers, if there is a need to address multiple sensors at once, but one has to note that if higher sensitivity is required, lower data rates must be used for the WUR (1–5 kb/s), so the wake up packets can be longer in time compared to other WBAN communications.

As mentioned, this mode of operation can be realised in higher layers of the communication protocols, and can be MAC protocol independent. The WUR is used to instantly transition the sensor node (with very low latency) from one mode of operation to another. This can be performed in a more energy-efficient manner with a WUR than using just the regular protocol, because most of the communication protocols, if required to work with low latency, require significant amount of power to be spent on beacon or connection events.

## 6. Proposed MAC Protocol for Wireless Healthcare Environments—WhMAC

Considering the presented network architecture, there is the possibility of lowering the amount of unnecessary overhead and idle listening in order to achieve efficient wireless communications in health measurement applications. The idea is to use a very simple, but efficient, TDMA MAC strategy and use the MN for coordinating the synchronisation, wake-up signals and data transmission to the monitoring stations.

We integrated the developed protocol described in [27] with the WUR in order to obtain a WUR-enabled wireless healthcare MAC protocol (WhMAC) for efficient stream data transfer and efficient infrequent monitoring for a WBAN network. Protocol [27] is an energy-efficient MAC protocol suitable for communication in a Wireless Body Area Network. The protocol takes advantage of the fixed nature of the Body Area Network to implement a TDMA strategy with very little communication overhead, long sleep times for the sensor transceivers and robustness to communication errors. Simulations have been done to determine this robustness. The protocol is implemented on the Analog Devices ADF7020 RF transceivers for which power modelling and estimations have been done. This model will be extended with the WUR to accurately predict the energy consumption.

### 6.1. TDMA Frame, Packet and Wake-Up

**Figure 4.**
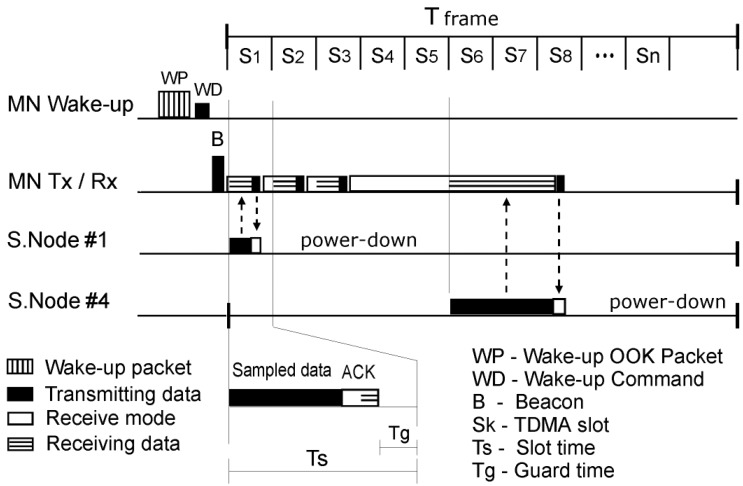
whMAC TDMA frame.

The whMAC TDMA frame is presented in Figure 4. It consists of a beacon (B), sent by MN, and a number of time slots. Every measured quantity is assigned to one or more time slots, depending on its size. A single value, such as heart-rate, or blood pressure can be sent using only one time slot, and hence it is given only one time slot. On the other hand, streaming of ECG needs multiple time slots in order to achieve the required data-rate. Some slots can be also merged and the packet can be sent across all of them to reduce overhead. Some reserve time slots should be left for a real-time data stream, in the case of errors occurring. The decision on what quantities are going to be measured is made by the MN, using the predefined scenarios, or can be selected using the MN or MS. For example, if the patient has flu, the slot “S1” could be used for temperature, “S2” for blood pressure and so on. In another example, if the patient needs to be monitored for ECG, for example “S1” would be heart rate, and “S2–12” would be used for the ECG data stream. Every measured quantity should also be associated with a preferred sensor, and if possible, a backup sensor (or sensors).

**Figure 5.**
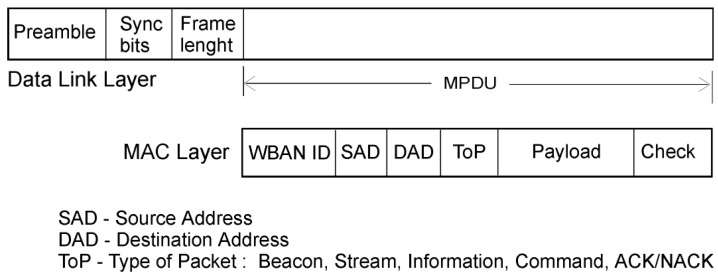
whMAC data packet: wake up command, beacon, data, status and ACK/NACK packets.

The whMAC communication packet is shown in Figure 5. Its MAC layer consists of:

- *WBAN ID*—WBAN ID, given during network forming.
- *Source Address*—Sensor or MN address
- *Destination Address*—Sensor, MN or broadcast address
- *Type of packet*—Beacon, Stream, Information, Commands, ACK/NACK
- *Payload*—Sampled data or additional information
- *Check*—CRC or FEC

These communication packets can be of different size, depending on the type. For example, information packets sent in one time slot can be shorter than the stream packets, sent across multiple time slots, to reduce overhead.

**Figure 6.**
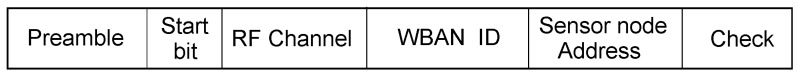
whMAC wake up packet: OOK modulated packet.

A whMAC wake-up packet can be seen in Figure 6. It consists of:

- *Preamble*—used to generate the wake-up signal
- *Frequency channel*—Channel in which to expect a command.
- *WBAN ID*—WBAN ID, given during network forming.
- Sensor address
- *Error detection*—CRC or FEC

The wake-up packet is always followed by a communication command packet, intended for the same address, at the frequency channel sent in the wake-up packet. The wake-up communication has its own carrier frequency and it cannot use multiple channels. As for the timing of the wake up packet, it is important to be as close to the end of the time frame (to be followed by the beacon), in order to reduce idle listening for a beacon to synchronise. Therefore, at the end of the time frame, certain number of time slots should be freed if needed for wake up packets.

### 6.2. Addressing

Every sensor would have its own identifier address based on its function, which would be determined by the standard (for example a temperature sensor has address 1, SPO2 has 2, ECG has 3, *etc.*). There might also be a group of sensors of the same type (for example accelerometers), and they should have unique identifier based on the position on the body. Also, identifiers would be different if a sensor is “simple” and only sends raw data, or if it is DSP enabled. Therefore, the address should describe: (1) Type of the sensor; (2) Position on the body; (3) Standardised sensor characteristics. Since medical equipment has to be standardised anyway, this wireless address standardisation should not be difficult to achieve.

### 6.3. Modes of Operation

As mentioned earlier, every time slot has its value assigned to it, and the preferred sensor to obtain it. For example, if pulse is needed, the MN assigns a time slot for all communication regarding this value. Then it sends a wake up signal and a command to the address that is used by the SPO2 sensor to measure the pulse. If the sensor does not respond, the MN addresses alternate sensor (e.g., DSP enabled ECG), asking for the same value, before eventually concluding that signal is unavailable. Also, if streaming of the data is necessary, the MN addresses the sensor and gives it a number of time slots, depending on the data rate and required latency (generally, it would advocate the number of time slots for streaming, and some reserve time slots, in case of data loss). After this is done, streaming works as described in [27], and further commands are provided using ACK/NACK packets.

### 6.4. Frequency Considerations

Once a MN starts forming a network, it chooses a channel randomly and listens for a beacon. The beacon signal is sent using higher power than intra-BAN communication, and it is used for TDMA synchronising and MN-to-MN localisation. In general, it should be of higher range than intra-BAN communication. If a channel is free, the MN starts sending beacons, looks at the desired scenario and sends wake-up signals to desired sensors, with command packets using the free frequency for connection. Of course, if nearby sensors are connected to a different patient they should reject the wake-up signals with different WBAN IDs if they detect them. After this, the sensors are in a “stand-by” mode, paired with the MN, waiting for a new wake up signal to change the state if needed.

If there is a need for constant monitoring, the MN occupies a free channel, and the patient is usually immobilised to get clear signals. From then, on a regular basis, or if packet error increases, the MN should stop TDMA and check the channel. If it detects that beacons from another MN on the same channel are getting stronger (another WBAN on the same frequency is approaching), the frequency should be changed to a random, free channel, using WUp + command. Additionally if there is an emergency situation, there should be free channels that are dedicated only for this types of situations.

### 6.5. Timing Considerations

TDMA frame and slot timings are determined by communication data rates and packet size, which are determined by the sample rates. For example, if the communication data rate is *f_c_* bit/s and the status packet takes a maximum of *n* bits in total, the duration of the time slot does not need to be longer than *T_s_* = *n/f_c_* + *T_guard_* for optimal channel utilisation. *T_guard_* is the guard time and it is determined by clock accuracy and resynchronisation time. On the other hand, the number of time slots needed for streaming is determined by the sample rate, data rate and TDMA frame. The guard time is necessary to avoid overlapping of the transmissions from different sensors due to clock drift. Usually there would be more than one packet per TDMA frame for streaming, depending on BER (bit error rate) and packet overhead, for optimal communication. Also, when slots are assigned, there should be some free time slots left in reserve, in case of the errors. This is especially important in case of the real-time streaming.

On the other hand, it has to be noted that wake up receiver communication would work at a lower data rate than normal communication, in order to achieve low power. For example, the wake up receiver would work with data rate of 5 kb/s to 10 kb/s, which is significantly lower than the transceivers data rate of for example 200 kb/s. This means that even if the wake up packet has less bits than the communication packet, it will probably take more time to be transmitted. Therefore, if the same transceiver is used to send both types of packets, the wake up packet could take more time slots.

### 6.6. whMAC Power Consumption Model

When a power consumption model for communication needs to be built, duty cycle needs to be considered first. Duty cycle is calculated as the fraction of time that a system is in an “active” state. For the sensor transceiver, this is the time the transceiver is on (RF activity time), regardless of whether it is transmitting data, receiving data or idly listening to a clear channel. Low power protocols are developed to reduce these times so that the transceiver can be switched off for longer durations of time. Introducing the wake up receiver as a component that can listen to a channel reduces this duty cycle (time spent on idle listening), but introduces new component with quiescent power consumption. Good power model must be derived to justify the introduction of this component and determine the maximum power consumption it can have in order to be practical in applications.

For wake up packets, the energy used by the sensor for receiving one wake up signal is calculated as:

(1) $$E_{\text{wup}}=P_{wd}T_{\text{wup}}\;(1+\text{wer}+\text{per})+\;P_{R}T_{\text{cmd}}\;(1+\text{per})$$

*P_wd_* and *T_wup_* are the dynamic power consumed by the wake up receiver while receiving a wake up packet, and its duration, respectively. *P_R_* is average power while receiving a packet (command) and *T_cmd_* is the duration of the command. *wer* and *per* are error rates for the wake up and data packets. The energy consumption of the sensor for sending one status packet can be calculated as:

(2) $$E_{\text{stat}}=(P_{R}T_{\text{beac}}+P_{T}T_{\text{stat}}+P_{R}T_{\text{ack}}\;)\;(1+\text{per})$$

*P_T_* is average power while sending data (status packet). *P_R_* is average power while receiving packet (beacon or acknowledgement). *T_beac_*, *T_stat_* and *T_ack_* are the durations of beacon, status and acknowledgement packets respectively.

Streaming of data is done by assigning a number of time slots for sensor that streams data. This is a power-hungry mode, where every little increase in overhead or idle listening significantly increases power consumption, due to the amount of data being sent. We have no idle listening when this method is used for streaming. Some methods of reducing this energy are:

1. Reducing the overhead by increasing the size of the packets.
2. Increasing the data rate.

Increasing the packet size would increase the packet error rate, and for a given Bit Error Rate (BER), the optimal packet size can be calculated. Also, while streaming, the sensor does not need to listen the beacon in every time slot to resynchronise and commands can be given with the ACK. It can wait for *N_R_* time slots before resynchronising to save power. *N_R_* is calculated as:

(3) $$N_{R}\leq \frac{1}{2}\left(\frac{T_{g}}{T_{\text{frame}}\theta }\right)$$

where *T_g_* is guard time, and *θ* is clock accuracy. Typical value for *N_R_* would be 30 for *T_frame_* = 1 s, *θ* = 30 ppm and *T_g_* = 2 ms.

The energy used for streaming is then given by:

(4) $$E_{\text{stream}}=\left(\frac{P_{R}T_{\text{beac}}}{N_{R}}+P_{T}T_{\text{data}}+P_{R}T_{\text{ack}}\right)\;(1+\text{per})$$

where *P_R_* is power spent while receiving beacon (it is divided by *N_R_*), and receiving the acknowledgement. *P_T_* is the power while sending the data packet. *T_beac_T_data_T_ack_* are the durations of the beacon, data and ACK packets respectively.

Therefore, the total power overhead for communication during time while sensor is in a WBAN is calculated as:

(5) $$P_{\text{total}}=P_{ws}+\frac{n_{\text{wup}}E_{\text{wup}}+n_{\text{stat}}E_{\text{stat}}+\frac{T_{\text{stream}}}{T_{\text{frame}}}E_{\text{stream}}}{T_{on}}$$

*P_ws_* is the quiescent power consumption of the wake up receiver. *n_wup_* and *n_stat_* are the numbers of wake up commands and status frames in the period where we measure power consumption (*T_on_*). *T_stream_* is the time spent on streaming data (the duration of the monitored signal). The idea is to have a function *P_total_* = *f*(*n_wup_*, *n_stat_*, *T_stream_*, *T_on_*), where the input parameters would be application dependent, and everything else would be hardware dependent (dependent on data rate, hardware power consumption, sample rates, *etc.*)

For the power consumption, we will consider an application with real components, and discuss the power consumption. The architecture of the sensor and MN is shown in Figure 2. We will look at the sensor modes of operation. The power consumption for each mode is calculated as:

- *Disconnected and Stand by*—The sensor is off, waiting for a command. *P_sb_* = *P_ws_*
- *Monitoring*—The sensor is waiting for a command, and woken up to send measured data with the frequency *f_m_*:
  $$P_{\text{mon}}=P_{ws}+f_{m}\;(E_{\text{wup}}+E_{\text{stat}})$$
- *Stream*—The sensor is woken up to stream data for the duration of *T_stream_*.
  $$P_{\text{str}}=P_{ws}+E_{\text{wup}}/T_{\text{stream}}+E_{\text{stream}}/T_{\text{frame}}$$

For example, we will consider ADF7020 transceiver for data transfer, MSP430 microcontroller, and our added wake up circuit. For our wake up receiver, the relevant values are given in Table 1.

Timing values for the presented packet formats are: *T_wup_* = 8.7*ms*, *T_cmd_* = 1.08 ms, *T_stat_* = 1.08 ms, *T_beac_* = 0.44 ms and *T_ack_* = 0.44 ms. We will consider for streaming a signal sampled with stream data rate of *f_s_*, which would give various *T_data_* for a *T_frame_* = 1 s. For this example, the power used by the sensor for communication is given in Table 2.

In a typical application, depending on the type of the sensor used, the sensor can go through various states, and using the derived model for the hardware, the energy consumption can be predicted. Also, some sensors can be in two modes, streaming and monitoring, where additional power must be added.

### 6.7. Full Application Power Analysis and Comparisons

**Figure 7.**
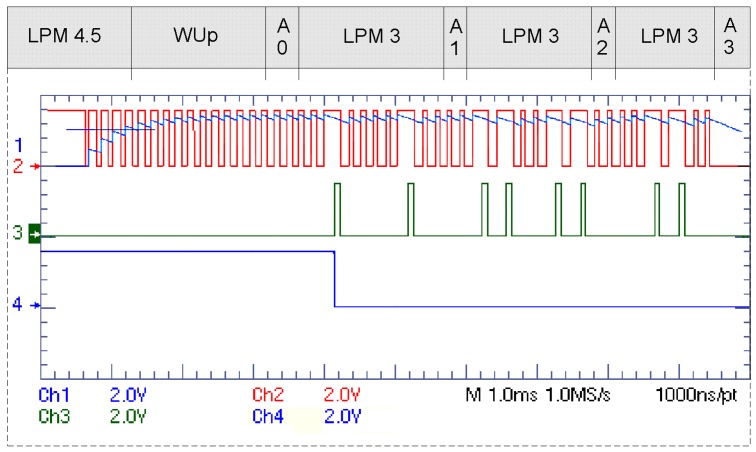
Measured wake up signal (1), SPI clock (2), SPI data (3), SPI enable (4) and MSP430F5x power modes while receiving a wake up packet.

For the whole application, we also have to take into account the communication power and the effect that the protocol has on the microcontroller power consumption. In any low power application, the sleep modes are used for the microcontroller when it is not needed in order to save power. The more the protocol needs the microcontroller's resources, the higher effect it has on the communication power consumption, depending on the type of the microcontroller. This becomes very important when we have long sleeping intervals and the whole sensor could be switched off. Even the type of Low Power Mode is important in which the microcontroller awaits the wake up signal, or processes it. For example, in Figure 7 it is shown how for our wake up receiver, the microcontroller can be in shut-down mode until it gets a wake up signal, and then with a few very short active modes, processes it. This is very important, because wake up receivers would not have good filtering and multi-frequency operations, and the sensors would overhear signals that are not intended for them. Table 3 shows the MSP430 power modes while receiving a packet.

We will give comparisons for two variants of the protocol used. Using the whMAC, and using the protocol [27] as the lowest power consumption protocol without wake up receivers. Comparison will be made for signals that need to be measured regularly, but with high monitoring intervals (non-stream). For streaming data, the power consumption would be equal.

**Figure 8.**
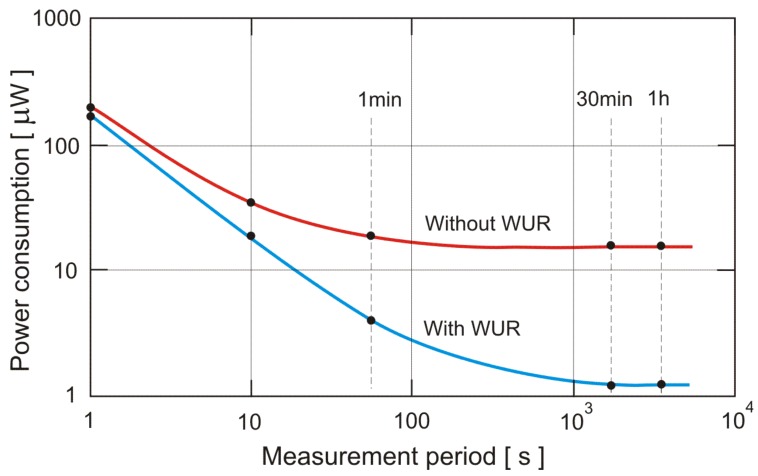
Power consumption with and without WUR.

For the example of MSP430F5xx microcontroller, an ADF7020 transceiver and the developed WUR, and considering constant monitoring of periods from 1 h to 1 s (measurement periods), the power consumption comparisons are given in Figure 8.

For on-demand events, an accurate power comparison cannot be made, and one has to consider also the high latency which will be present if the wake up receiver is not used. For the example we have given in the Table 4, the maximum latency would be 20 s. On the other hand, if one wishes the same latency without using a wake up receiver, the power consumption will be significantly higher than if wake up receiver is used, regardless of the protocol. Table 4 also gives the break-down of the power consumption and energy of the various devices as given in Figure 2 for different average communication intervals (measurement periods). If the average communication interval is 1 s, the calculated power consumption is equivalent to streaming of data in the TDMA protocol with a frame time of 1 s.

## 7. Conclusions

mHealth, enabled by mobile phones and other wireless computing devices, is promising to bring a revolutionary adoption of new communication patterns in healthcare. The non-intrusive health monitoring of patient's vital signs over wireless body area networks (WBANs) provides a cost-effective solution to the healthcare system. Currently, WBAN technology is being widely investigated and is still in its primitive stage. This technology could lead to the realisation of such concepts as mHealth in the future. However, before the WBAN can be widely used in long term monitoring of the patients' health, one needs to address the difficult power consumption constraints. In this work we presented some novel approaches for power optimisation within a WBAN. A novel, signal-oriented WBAN approach is presented, and the method of standardising the WUR in WBAN environment is investigated. Finally we propose a WhMAC protocol, which is a WUR enabled—signal oriented low power protocol, intended to be used for physiological signal monitoring in medical or home settings, for both high data rate or low data rate sensor applications.

As the WUR enables a power-efficient event-driven wireless environment, it is possible to turn physiological data into medical information through the provision of an accurate, reliable and very low power WBAN in-network processing solution for respiration rate, heart rate, pulse, blood oxygen level measurements using off-the-shelf ECG, SpO2 and accelerometer sensors. One of the possible implementations of the protocol can be an E-MEWS system (wireless electronic early warning score in medical admissions) [28], where some health parameters are extracted at regular time intervals, and depending on their values, the patient's health condition is graded from normal to critical. Further usage can be for home monitoring of a patient, if the sensors are small enough to be worn for a long period of time.

Although primarily aimed at medical devices, the proposed system can also be used in consumer healthcare, sports and fitness applications, the home care and elderly assisted living, entertainment, interactive gaming, smart power supplies, energy harvesters, *etc.*

## Figures and Tables

**Table 1.** ADF7020 and Wake Up Receiver characteristics.

**Communication Parameters**	**Measured Values**
***ADF7020 transceiver***	
Communication band—ISM	433 MHz
Communication data rate	200 kb/s
RF output power	−10 dBm
Low Power Sleep Mode	230 nW
Consumption in receive mode	P_R_ = 57 mW
Consumption in transmit mode	P_T_ = 47.7 mW

***Wake up receiver (WUR)*** [2]	
Data rate	5.5 kb/s
Quiescent power	P_ws_ = 270 nW
Receiving-Dynamic power	P_wd_ = 3 *μ*W

**Table 2.** Power consumption values.

***Disconnected and Stand-by***	
WUR static power	P_sb_ = 270 nW
***Monitoring***	
*Monitoring period*	*Communication power*
T_m_ = 1 h	P_mon_ = 0.315 *μ*W
T_m_ = 30 min	P_mon_ = 0.36 *μ*W
T_m_ = 1 min	P_mon_ = 3 *μ*W
T_m_ = 10 s	P_mon_ = 16.5 *μ*W
T_m_ = 1 s	P_mon_ = 163 *μ*W

***Stream (calculated using [***27***])***	
*Sample size and rate*	*Communication power*
12 bit sample @ 128 Hz	P_str_ = 0.52 mW
16 bit sample @ 128 Hz	P_str_ = 0.64 mW
16 bit sample @ 256 Hz	P_str_ = 1.09 mW

**Table 3.** MSP430 Power Modes from Figure 7.

**Abbreviation**	**Description**
LPM 4.5	Shut-down mode
WUp	Wake up time from LPM 4.5 to active state
LPM 3	Low power mode with operational SPI slave mode and timer/counters
A0	Active mode for initialisation
A1, A2, A3	Active modes for processing 3 wake up packet bytes

**Table 4.** Protocol Comparisons.

**Power Consumption Constituents**	**Measurement period**				

	**1 h**	**30 min**	**1 min**	**10 s**	**1 s**
1 Data transmission power	0.045	0.09	2.73	16.23	162.73
2 Microcontroller power	0.007	0.014	0.41	2.44	24.5
3 WUR power	0.27	0.27	0.27	0.27	0.27
4 ADF7020 OFF mode power	0.3	0.3	0.3	0.3	0.3
5 MSP430F5xx LPM 4.5	0.52	0.52	0.52	0.52	0.52
6 MSP430F5xx LPM 3	7.8	7.8	7.8	7.8	7.8
7 ADF7020 and MSP430 TDMA power	7.85	7.85	7.85	7.85	7.85

A whMAC (1 + 2 + 3 + 4 + 5)	1.142	1.194	4.23	19.76	188.32
B Protocol [27] (1 + 2 + 4 + 6 + 7)	16.002	16.054	19.09	34.62	203.18

C Power reduction coefficient	14.01	13.45	4.51	1.75	1.08

* Values for rows 1–7 and rows A and B are in *μ*W.
